# COVID-19 outcomes of 10,881 patients: retrospective study of neurological symptoms and associated manifestations (Philippine CORONA Study)

**DOI:** 10.1007/s00702-021-02400-5

**Published:** 2021-08-27

**Authors:** Adrian I. Espiritu, Marie Charmaine C. Sy, Veeda Michelle M. Anlacan, Roland Dominic G. Jamora, the Philippine CORONA Study Group Investigators, Corina Maria Socorro A. Macalintal, Joanne B. Robles, Paulo L. Cataniag, Manolo Kristoffer C. Flores, Noreen Jhoanna C. Tangcuangco-Trinidad, Dan Neftalie A. Juangco, Giuliani Renz G. Paas, Audrey Marie U. Chua, Valmarie S. Estrada, Philip Rico P. Mejia, Therese Franz B. Reyes, Maria Teresa A. Cañete, Ferdinand Renfred A. Zapata, Franko Eugenio B. Castillo, Romulo U. Esagunde, Jean B. Gantioque, Maritoni C. Abbariao, Geramie M. Acebuque, Evram V. Corral, Marian Irene C. Escasura, Marissa T. Ong, Krizelle Cleo Fowler, Arnold Angelo M. Pineda, Khasmeen D. Aradani, Joseree-Ann S. Catindig, Mark Timothy T. Cinco, Mark Erving H. Ramos, Romulus Emmanuel H. Cruz, Marita B. Dantes, Norberto A. Francisco, Rosalia A. Teleg, Krisverlyn B. Bellosillo, Jean Paolo M. Delfino, Cid Czarina E. Diesta, Rosalina B. Espiritu-Picar, Julie Anne V. Gamboa, Cara Camille M. Matute, Franzelle P. Padilla, John Joshua Q. Punsalan, Ma. Epifania V. Collantes, Charmaine B. Que, Hanifa A. Sampao, Maxine Camela S. Sta. Maria, Marita M. Fuentes, Jennifer Justice F. Manzano, Rizza J. Umali, Marc Conrad C. Molina, Hazel Claire Minerva-Ang, Arturo F. Surdilla, Loreto P. Talabucon, Natasha F. Wabe, Maria Victoria G. Manuel, Al Inde John A. Pajantoy, Josephine Cecilia V. Roque, Paul Emmanuel L. Yambao, Christian Paul B. Banday, Chritopher C. Cipriano, Nehar A. Pangandaman, Avery Gail C. Wasil, Elrey P. Inocian, Jarungchai Anton S. Vatanagul, Almira Doreen Abigail O. Apor, Carissa Paz C. Dioquino, Prinz Andrew M. Dela Cruz, Maricar P. Yumul, Ma. Alma E. Carandang-Concepcion, Ma. Caridad V. Desquitado, Carl Kevin L. Julao, Dante P. Bornales, Generaldo D. Maylem, Mark Joseph F. Cuntapay, Annabelle Y. Lao-Reyes, Aileen Mae B. Lee, Nadia O. Manlegro, Dave Mar L. Pelere, Lina C. Laxamana, Diana-Lynn S. Que, Jeryl Ritzi T. Yu, Ma. Socorro C. Martinez, Alexandria E. Matic, John Angelo Luigi S. Perez, Glenn Anthony A. Constantino, Aldanica R. Olano, Liz Edenberg P. Quiles, Artemio A. Roxas, Jo Ann R. Soliven, Michael Dorothy Frances Montojo-Tamayo, Ma. Lourdes C. Joson, Jojo R. Evangelista, Ma. Clarissa B. Nuñez, Marietta C. Olaivar, Dominique Q. Perez, Mark Deneb O. Armeña, Robert A. Barja, Joshua Emmanuel E. Abejero, Maritzie R. Eribal, Ryndell G. Alava, Muktader A. Kalbi, Nasheera W. Radja, Mohammad Elshad S. Sali

**Affiliations:** 1 Division of Adult Neurology, Office of the Department of Neurosciences, College of Medicine and Philippine General Hospital, University of the Philippines Manila, Taft Avenue, Ermita, 1000 Manila, Philippines; 2 Department of Clinical Epidemiology, College of Medicine, University of the Philippines Manila, Manila, Philippines

**Keywords:** COVID-19, New-onset neurological symptoms, Mortality, Respiratory failure, Intensive care unit admission, Cohort study

## Abstract

Our study aimed to determine the effects of new-onset neurological symptoms (NNS) on clinically relevant outcomes in hospitalized patients with COVID-19 infection. We conducted a nationwide, comparative, retrospective, cohort study among adult, hospitalized COVID-19 patients involving 37 hospital sites from various regions in the Philippines. We included a total of 10,881 patients with confirmed COVID-19 infection (2008 had NNS while 8873 did not have NNS). The adjusted hazard ratios (aHRs) for mortality among the mild and severe cases were significantly higher by 1.660 (95% CI 1.132–2.435) and by 1.352 (95% CI 1.042–1.752), respectively, in the NNS group compared to those in the non-NNS group. The aHRs for respiratory failure in the NNS group were significantly increased by 1.914 (95% CI 1.346–2.722), by 1.614 (95% CI 1.260–2.068), and by 1.234 (95% CI 1.089–1.398) among the mild, severe, and critical cases, respectively. The aHRs for ICU admission in the NNS group were still significantly higher by 1.973 (95% CI 1.457–2.673) and by 1.831 (95% CI 1.506–2.226) among the mild and severe cases, respectively. Patients who had NNS were not significantly associated with a longer duration of ventilator dependence (adjusted odds ratio (aOR) 0.954, 95% CI 0.772–1.179), longer ICU stay (aOR 0.983, 95% CI 0.772–1.252) and longer hospital admission (aOR 1.045, 95% CI 0.947–1.153). The presence of NNS significantly increases the risk of mortality, respiratory failure and ICU admission among COVID-19 patients. Registration and associated protocol publication: ClinicalTrials.gov website (NCT04386083); Espiritu AI, Sy MCC, Anlacan VMM, Jamora RDG. The Philippine COVID-19 Outcomes: a Retrospective study Of Neurological manifestations and Associated symptoms (The Philippine CORONA study): a protocol study. BMJ Open. 2020;10:e040944.

## Introduction

The coronavirus disease 2019 (COVID-19) is caused by severe acute respiratory syndrome coronavirus 2 (SARS-CoV-2) and has affected over 192 million individuals worldwide as of July 23, 2021 (Coronavirus disease (COVID-19) n.d.). During this period in the Philippines, our data breached the 1.5 million mark of confirmed total cases; nearly 54 thousand are active cases and approximately 28 thousand patients died from this infection (Department of Health (Philippines) 2021). Nationwide, about 50% of the intensive care unit (ICU), isolation, and ward beds were occupied and nearly 40% of the total mechanical ventilators were used (Department of Health (Philippines) 2021). In July 2021, only 5.5 million individuals, approximately 5% of the population, were fully vaccinated in the country (Coronavirus (COVID-19) Vaccinations–Statistics and Research–Our World in Data 2021), which is exceedingly distant from the target of about 75–90% vaccinated individuals to achieve herd immunity from this infection (Anderson et al. 2020). With the recent advent of local transmission of the Delta (B.1.617.2) variant in the country which could potentially initiate a surge and overwhelm our healthcare systems, the Department of Health is currently focusing its attention to implement stricter border control policies and to strengthen local COVID-19 responses (Department of Health (Philippines) 2021).

Current reports of neurological symptoms/signs and complications of this infection are limited due to the small number of included patients and relatively short duration of data collection which could hinder more precise estimates and detection of rarer manifestations (Amanat et al. 2021; Benussi et al. 2020; Chachkhiani et al. 2020; Chuang et al. 2021; Collantes et al. 2021; Flores-Silva et al. 2021). Although there were two powered studies that provided a glimpse on the effects of neurological manifestations on mortality (Eskandar et al. 2021; Frontera et al. 2021), there are no comparative cohort studies yet that explored the effects of new-onset neurological symptoms (NNS) on other clinically relevant outcomes such as respiratory failure, duration of ventilator dependence, admission to the intensive care (ICU) unit and length of ICU and hospital stay. Furthermore, these two large studies were conducted in New York City (NYC) where healthcare systems and outcomes may be remarkably different from low–middle income or developing countries like the Philippines.

Therefore, we aimed to determine the clinical/neurological features of hospitalized patients with COVID-19 infection and to investigate the effects of NNS on mortality, respiratory failure, duration of ventilator dependence, ICU admission, length of ICU and hospital stay among these patients.

## Methodology

### Study design

We performed a nationwide, multicenter, comparative, retrospective, cohort study involving patients with COVID-19 who were admitted to our hospitals/study sites from February 2020 until December 2020. The study’s protocol was registered in ClinicalTrials.gov (NCT04386083) and was previously published (Espiritu et al. 2020).

### Setting

The study encompassed a total of 37 major hospitals/study sites from various regions in the Philippines (see the complete list of the sites below).

### Patient selection, sampling and cohort description

We included a total enumeration of all patients that fulfilled the inclusion criteria, as follows: adults ≥ 19 years of age; confirmed cases via COVID-19 real-time reverse transcription polymerase chain reaction (RT-PCR) of patients’ nasopharyngeal swab samples which were performed by testing centers accredited by the Department of Health (Philippines); clinical symptoms/signs ascribed to COVID-19 infection; patients with the appropriate disposition by the end of the data collection period (e.g., discharged, transferred to another hospital, or died). Individuals who were transferred to another hospital were excluded to prevent duplication of data.

Adult COVID-19 patients who had new-onset neurological symptom/s (NNS) were grouped under the exposed cohort while those without NNS (non-NNS) were classified under the unexposed cohort.

### Information sources, data collection, patient variables, and bias

We collected relevant information from the patient medical charts. Pilot-tested electronic collection forms generated using Epi Info Software (Version 7.2.2.16) were employed. The details of the obtained patient variables were indicated in the published protocol (Espiritu et al. 2020). Recording bias was considered inherent in this retrospective cohort study.

### Outcome variables

We obtained the following relevant patient outcomes: mortality; respiratory failure (patients with clinical symptoms/signs of respiratory insufficiency (increased work of breathing/tachypnea [respiratory rate of ≥ 22], a necessity to administer supplemental oxygen, or abnormal blood gases [Partial pressure of oxygen < 60/hypoxemia or partial pressure of carbon dioxide > 45/hypercapnia])); duration of ventilator dependence (DVD) (days from the start of assisted ventilation to cessation); ICU admission (COVID-19 patients admitted to an ICU or ICU-comparable setting; length of ICU stay (LICUS) (days admitted in the ICU); and length of hospital stay (LHS) (days from admission to discharge).

### Sample size

The calculation of the sample size, computed at 1342 patients, was specified in the published protocol (Espiritu et al. 2020).

### Statistical analysis

We summarized demographic, medical, and neurological characteristics using frequencies (%) for categorical variables and medians (interquartile range, IQR) for continuous variables. We determined distribution differences between two independent samples using Mann–Whitney *U* and *χ*^2^ tests. We employed odds ratios (OR) and hazard ratios (HR) and corresponding 95% confidence intervals (CI) as the outcome measures for our dichotomous and time-to-event outcomes, respectively. The ORs for longer ventilator dependence, longer ICU stay, and longer hospitals stays were computed via multivariate logistic regression. Data on the time to onset of mortality, respiratory failure, ICU admission were used to build Kaplan–Meier curves; the log-rank test was employed to compare the curves. Confounder and effect modifiers were identified by performing stratified analysis. Adjusted HRs and ORs with 95% CI were computed based on prespecified confounders/effect modifiers. Significant factors that affect the outcomes were determined using Cox proportional regression models. The final model was further rectified using likelihood ratio tests and other information criteria (Akaike or Bayesian). Goodness-of-fit was assessed via Hosmer–Lemeshow test and Cox–Snell residuals. A *p* value < 0.05 (two tailed) was set for all analyses. Statistically significant differences were also detected if the 95% CI did not include number one. We conducted all statistical analyses using Stata^®^, Version 7.2.2.16 (College Station, TX: StataCorp LP).

### Ethical considerations

This study was approved by the individual institutional review and research boards of the hospital sites and the Single Joint Research Ethics Board of the Department of Health of the Philippines (see complete list below).

## Results

### Inclusion of patients

**Fig. 1:**
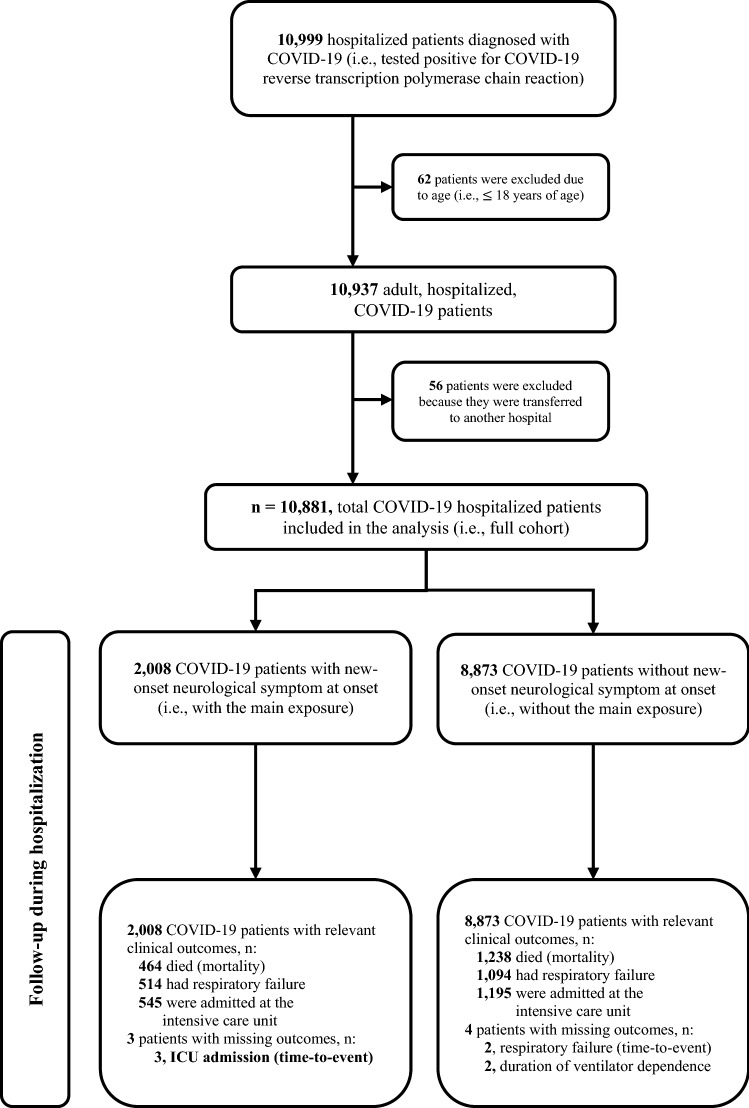
Flow of patients in the Philippine CORONA study

We identified 10,999 hospitalized patients diagnosed with COVID-19 (i.e., verified via RT-PCR) from the 37 participating study sites. A total of 10,881 were included in the qualitative and quantitative analyses. Two thousand eight patients were identified with the primary exposure (i.e., NNS group) while the remaining 8873 did not have the exposure (i.e., non-NNS group). Missingness of data for relevant outcomes was considered very minimal; thus, we deemed it unnecessary to explore its effects (see Fig. 1).

### Demographic and clinical characteristics of included COVID-19 patients

The median (IQR) age of the full cohort was 52 (36–64) with a female:male ratio was 1:1.13. A higher proportion of patients with NNS was found among those who are elderly (≥ 60 years) compared to non-elderly (19–59 years) (19.8% vs. 17.7%; *p* = 0.007). No significant difference in the proportion of patients with NNS was found between females and males (18.7% vs. 18.35; *p* = 0.609). The most typical declared exposure to COVID-19 infection was community/domestic travel (*n* = 3894, 35.8%). Hypertension (HPN) (*n* = 3647, 33.5%) and diabetes mellitus (DM) (*n* = 2191, 20.1%) were the most common comorbidities. Most patients had mild (*n* = 6690, 61.5%) and severe (*n* = 2354, 21.6%) COVID-19 infection. A considerable proportion of patients received systemic glucocorticoids (*n* = 2844, 26.1%), remdesivir (*n* = 1344, 12.4%), and tocilizumab (*n* = 1029, 9.4%). Other pertinent clinical features and comparison of characteristics of the NNS and non-NNS groups are displayed in Table 1.

**Table 1:** Demographics and clinical features of patients included in the analysis

Features	All patients	COVID-19 patients with NNS	COVID-19 patients without NNS	*p* value*
Sample, *n* (%)	10,881 (100.0)	2008 (18.4)	8873 (81.5)	–
Age in years, median (IQR)	52.0 (36.0–64.0)	54.0 (38.0–65.0)	52.0 (35.0–64.0)	< 0.001
Frequencies of age groups, *n* (%)				
60 years and above	3834 (35.2)	760 (37.8)	3074 (34.6)	0.007
19–59 years	7047 (64.8)	1248 (62.2)	5799 (65.4)	
Sex, *n* (%)				
Female	5099 (46.9)	951 (47.4)	4148 (46.7)	0.609
Male	5780 (53.1)	1056 (52.6)	4724 (53.2)	
Nationality, *n* (%)				
Filipino	10,789 (99.2)	1996 (99.4)	8793 (99.1)	0.179
Others	92 (0.8)	12 (0.6)	80 (0.9)	
History of COVID-19 exposure, *n* (%)				
International travel	323 (3.0)	42 (2.1)	281 (3.2)	< 0.001
Community/domestic travel	3894 (35.8)	871 (43.4)	3023 (34.1)	
Hospital	1362 (12.5)	266 (13.2)	1096 (12.4)	
Comorbidities, *n* (%)				
Hypertension	3647 (33.5)	927 (46.2)	2720 (30.6)	< 0.001
Diabetes mellitus	2191 (20.1)	523 (26.0)	1668 (18.8)	< 0.001
Kidney disease	611 (5.6)	174 (8.7)	437 (4.9)	< 0.001
Bronchial asthma	463 (4.2)	105 (5.2)	358 (4.0)	0.017
Coronary artery disease	421 (3.9)	100 (5.0)	321 (3.6)	0.004
Malignancy	244 (2.2)	70 (3.5)	174 (2.0)	< 0.001
Chronic obstructive pulmonary disease	156 (1.4)	24 (1.2)	132 (1.5)	0.32
Heart failure	127 (1.2)	44 (2.2)	83 (0.9)	< 0.001
Liver disease	60 (0.6)	17 (0.8)	43 (0.5)	0.048
Human immunodeficiency virus infection	37 (0.3)	9 (0.4)	28 (0.3)	0.357
Smoker, *n* (%)	1026 (9.4)	278 (13.8)	748 (8.4)	< 0.001
Healthcare worker, *n* (%)	876 (8.0)	235 (11.7)	641 (7.2)	< 0.001
Pregnant, *n* (%)	323 (3.0)	28 (1.4)	295 (3.3)	< 0.001
Respiratory and constitutional symptoms, *n* (%)				
Cough	4411 (40.5)	995 (49.6)	3416 (38.5)	< 0.001
Fever	3927 (36.1)	886 (44.1)	3041 (34.3)	< 0.001
Dyspnea	2703 (24.8)	613 (30.5)	2090 (23.6)	< 0.001
Sore throat	751 (6.9)	211 (10.5)	540 (6.1)	< 0.001
Fatigue	713 (6.6)	220 (11.0)	493 (5.6)	< 0.001
Sputum production	637 (5.8)	194 (9.7)	443 (5.0)	< 0.001
Rhinorrhea	607 (5.6)	200 (10.0)	407 (4.6)	< 0.001
Diarrhea	597 (5.5)	163 (8.1)	434 (4.9)	< 0.001
Arthralgia	175 (1.6)	45 (2.2)	130 (1.5)	0.013
Hemoptysis	33 (0.3)	6 (0.3)	27 (0.3)	0.968
COVID-19 disease severity, *n* (%)				
Mild	6690 (61.5)	1114 (55.5)	5576 (62.8)	< 0.001
Severe	2354 (21.6)	413 (20.6)	1941 (21.9)	
Critical	1707 (15.7)	468 (23.3)	1239 (14.0)	
Treatment received, *n* (%)				
Systematic glucocorticoids	2844 (26.1)	743 (37.0)	2101 (23.7)	< 0.001
Remdesivir	1344 (12.4)	325 (16.2)	1019 (11.5)	< 0.001
Tocilizumab	1029 (9.4)	188 (9.4)	841 (9.5)	0.873
Lopinavir–Ritonavir	579. (5.3)	99 (4.9)	480 (5.4)	0.387
Hydroxychloroquine	529 (4.9)	116 (5.8)	413 (4.6)	0.035
Chloroquine	358 (3.3)	68 (3.4)	290 (3.3)	0.789
Convalescent plasma	263 (2.4)	80 (4.0)	183 (2.1)	< 0.001

*NNS* new-onset neurological symptom, *IQR* interquartile range

*Difference between NNS and non-NNS groups

### Neurological features of included COVID-19 patients

The most common new-onset neurological symptom was headache (*n* = 607, 5.58%), anosmia/hyposmia (*n* = 544, 5.0%), and altered sensorium (*n* = 479, 4.4%). The proportion of patients who had new-onset neurological disorder/complication was 8.97% (*n* = 976); most patients had encephalopathy (*n* = 622, 5.72%), any acute cerebrovascular disease (*n* = 367, 3.37%), and any seizure event (*n* = 125, *n* = 1.16%). Among all the included patients, a number of individuals had a past neurological history of stroke/cerebrovascular diseases (*n* = 321, 2.95%), dementia (*n* = 38, 0.35%), and epilepsy (*n* = 27, 0.25%). Among patients who underwent computed tomography or magnetic resonance imaging (*n* = 760), most lesions were present in the parietal cortex (*n* = 113, 14.9%), basal ganglia (*n* = 112, 14.7%), and frontal cortex (*n* = 95, 12.5%). Very few patients in our cohort underwent cerebrospinal fluid analysis (*n* = 38). A more detailed presentation of neurological characteristics is displayed in Tables 2, 3.

**Table 2:** Past neurological history, and new-onset neurological symptoms and disorders/complications associated with the hospitalized COVID-19 patients included in the analysis (*N* = 10,881)

Past neurological history	Frequency (%)	New-onset neurological symptoms	Frequency (%)	New-onset neurological disorders/complications	Frequency (%)
Stroke/cerebrovascular diseases	321 (2.95)	Headache	607 (5.58)	Any neurological disorder/complication	976 (8.97)
Dementia	3 (0.35)	Anosmia/hyposmia	544 (5.00)	Encephalopathy	622 (5.72)
Epilepsy	27 (0.25)	Altered sensorium	479 (4.40)	Any acute cerebrovascular disease	367 (3.37)
Neuropathy	9 (0.08)	Ageusia/dysgeusia	338 (3.11)	Acute cerebrovascular infarction	262 (2.41)
Movement disorder	6 (0.06)	Myalgia	256 (2.35)	Acute cerebrovascular hemorrhagic stroke	101 (0.93)
Headache syndrome	5 (0.04)	Extremity weakness	246 (2.26)	Any seizure disorder	125 (1.16)
Central nervous system infection	5 (0.04)	Dizziness	159 (1.46)	Acute symptomatic seizure	63 (0.58)
Neuromuscular junction disorder	5 (0.04)	Confusion	143 (1.31)	Status epilepticus	19 (0.17)
Central demyelinating syndrome	2 (0.02)	Vomiting	126 (1.16)	Epilepsy	17 (0.16)
Myelopathy	2 (0.02)	Seizure	96 (0.88)	Anoxic brain	51 (0.47)
Peripheral nervoussystem infection	3 (0.03)	Dysarthria	83 (0.76)	Any movement disorder	3 (0.03)
Myopathy	0	Nausea	82 (0.75)	Inflammatory syndromes	
		Extremity numbness/paresthesia	53 (0.49)	Meningitis	13 (0.12)
		Facial weakness	40 (0.37)	Encephalitis	6 (0.06)
		Tremor	25 (0.23)	Meningoencephalitis	1 (0.01)
		Facial numbness/paresthesia	20 (0.18)	Acute disseminated encephalomyelitis	1 (0.01)
		Dysphagia	16 (0.15)	Acute necrotizing hemorrhagic encephalopathy	0
		Tongue weakness	8 (0.07)	Cerebellitis	0
		Blindness/decreased vision	6 (0.06)	Cerebellitis	0
		Ataxia	5 (0.04)	Optic neuritis	1 (0.01)
		Meningismus	5 (0.04)	Myelitis	0
		Hearing loss/decreased hearing	4 (0.04)	Sensory ganglionitis dorsal radiculitis	0
		Dysphonia	4 (0.04)	Anterior horn syndrome polio-like syndrome/ventral radiculitis	2 (0.02)
		Neck weakness	3 (0.03)	Peripheral neuritis/GBS-like syndrome	5 (0.04)
		Ophthalmoparesis/ophthalmoplegia	2 (0.02)	Peripheral neuritis other than GBS-like syndrome	1 (0.01)
		Eye pain	3 (0.03)	Myositis	1 (0.01)
		Bradykinesia	3 (0.03)	Neuromuscular disorder	3 (0.03)
		Dystonia	0		
		Choreoathethosis	0		

*GBS* Guillain–Barré syndrome

**Table 3:** Summary of findings in COVID-19 patients who underwent computed tomography/magnetic resonance imaging and cerebrospinal fluid (CSF) analysis

Findings	Frequency (%)
Radiologic imaging	
Number of patients with any radiologic imaging, *n*	760
Number of patients with the affected segment of the central nervous system evidenced by radiologic imaging, *n* (%)	
Whole brain	25 (3.3)
Frontal cortex	95 (12.5)
Temporal cortex	81 (10.6)
Parietal cortex	113 (14.9)
Occipital cortex	46 (6.0)
Subcortical white matter	90 (11.8)
Basal ganglia	112 (14.7)
Thalamus	44 (5.8)
Hypothalamus	3 (0.4)
Cerebellum	41 (5.4)
Midbrain	13 (1.7)
Pons	38 (5.0)
Medulla	2 (0.3)
Cervical spinal cord	3 (0.4)
Thoracic spinal cord	3 (0.4)
Lumbar spinal cord	3 (0.4)
Sacral spinal cord	0
CSF analysis	
Number of patients with CSF analysis, *n*	38
Median CSF total cell count (IQR), cells/μL	3.0 (2.3–14)
Number of patients with pleocytosis* in the CSF, *n* (%)	6 (15.8)
Median CSF neutrophil count (IQR), cells/μL	1 (0–8)
Number of patients with neutrophil/s^†^ in the CSF, *n* (%)	8 (21.0)
Median CSF lymphocyte count (IQR), cell/μL	13 (0–55)
Number of patients with lymphocytosis* in the CSF, *n* (%)	7 (18.4)
Median CSF protein (IQR), mg/dL	157.5 (37.8–708.0)
Number of patients with increased protein^‡^ in the CSF, *n* (%)	8 (21.0)
Median CSF glucose (IQR), mg/dL	7.6 (5.2–65.5)
Median serum glucose (IQR), mg/dL	126.0 (59.9–157.5)
Number of patients with hypoglycorrhachia^§^, *n* (%)	8 (21.0)

*IQR* Interquartile range

* > 5 cells/μL of CSF

^†^ ≥ 1 cell/μL of CSF

^‡^CSF protein concentration ≥ 60 mg/dL

^§^CSF glucose concentration < 2/3 of serum glucose concentration

### Effects of new-onset neurological symptom/s on outcomes of included COVID-19 patients and survival analysis

**Fig. 2:**
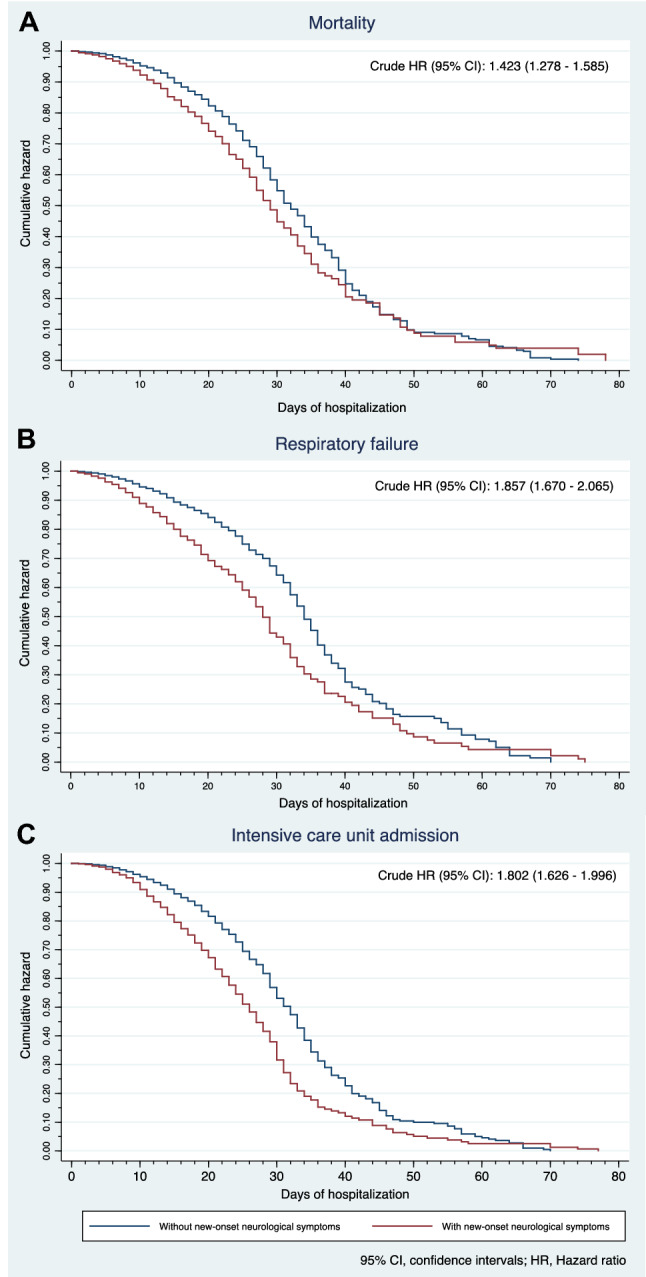
Cumulative hazard by exposure to new-onset neurological symptoms for **A** mortality, **B** intensive care unit admission and **C** respiratory failure

Table 4 shows the comparison of outcomes between NNS and non-NNS groups among the included COVID-19 patients while Table 5 presents the crude and adjusted hazard ratio for mortality, respiratory failure and ICU admission. Figure 2 shows the Kaplan–Meier cumulative hazard functions for NNS and non-NNS groups in terms of mortality, respiratory failure and ICU admission. For all survival analyses, it was found that the effect of NNS on the outcomes was significantly different depending on COVID-19 severity.

**Table 4:** Comparison of outcomes in COVID-19 patients with NNS vs. without NNS

Outcomes	All COVD-19 patients	COVID-19 patients with NNS	COVID-19 patients without NNS	*p* value*
Mortality and associated causes				
Mortality, *n* (%)	1702 (15.6)	464 (23.1)	1238 (14.0)	< 0.001^†^
Acute respiratory distress syndrome	749 (44.0)	216 (10.8)	533 (6.0)	< 0.001^†^
Septic shock	699 (41.1)	162 (8.1)	537 (6.1)	0.001^†^
Multi-organ dysfunction syndrome	150 (8.8)	23 (1.1)	127 (1.4)	0.321
Acute coronary syndrome	138 (8.1)	33 (1.6)	105 (1.2)	0.096
Cardiac arrhythmia	106 (6.2)	19 (0.9)	87 (1.0)	0.888
Brain herniation	66 (3.9)	43 (2.1)	23 (0.3)	< 0.001^†^
Decompensated heart failure	44 (2.6)	9 (0.4)	35 (0.4)	0.732
Respiratory failure and associated causes				
Respiratory failure, *n* (%)	1608 (14.8)	514 (25.6)	1094 (12.3)	< 0.001^†^
Pneumonia	927 (57.6)	324 (16.1)	603 (6.8)	< 0.001^†^
Acute respiratory distress syndrome	868 (54.0)	218 (10.9)	650 (7.3)	< 0.001^†^
Shock	147 (9.1)	48 (2.4)	99 (1.1)	< 0.001^†^
Central neurological cause	89 (5.5)	82 (4.1)	7 (0.1)	< 0.001^†^
Pulmonary edema	33 (2.0)	16 (0.8)	17 (0.2)	< 0.001^†^
Pulmonary embolism	21 (1.3)	2 (0.1)	19 (0.2)	0.404
Peripheral neurological cause	2 (0.1)	0	2 (0.02)	1.000
Pulmonary hemorrhage	1 (0.1)	1 (0.05)	0	0.185
Duration of ventilator dependence				
Duration of ventilator dependence				
Sample, *n*	1606	514	1092	0.612
Median (IQR)	13 (8–20)	13 (8–20)	13 (8–20)	
ICU admission and reasons for admission				
ICU admission, *n* (%)	1740 (16.0)	545 (27.1)	1195 (13.5)	< 0.001^†^
Acute respiratory distress syndrome	956 (54.9)	236 (11.8)	720 (8.1)	< 0.001^†^
Shock	180 (10.3)	59 (2.9)	121 (1.4)	< 0.001^†^
Impaired level of consciousness	110 (6.3)	85 (4.2)	25 (0.3)	< 0.001^†^
Acute myocardial infarction	80 (4.6)	13 (0.6)	67 (0.8)	0.610
Acute kidney injury necessitating dialysis	75 (4.3)	27 (1.3)	48 (0.5)	< 0.001^†^
Treatment-related indication	71 (4.1)	28 (1.4)	43 (0.5)	< 0.001^†^
Acute stroke	57 (3.3)	51 (2.5)	6 (0.1)	< 0.001^†^
Cardiac arrhythmia	36 (2.1)	6 (0.3)	30 (0.3)	0.782
Post-cardiac arrest	23 (1.3)	5 (0.2)	18 (0.2)	0.599
Cerebral edema	18 (1.0)	15 (0.7)	3 (0.03)	< 0.001^†^
Venous thromboembolism	13 (0.7)	1 (0.05)	12 (0.1)	0.484
Length ICU stay				
Length of ICU stay				
Sample, *n*	1737	545	1192	0.887
Median (IQR)	15 (9.5–21)	15 (10–21)	15 (9–21)	
Length of hospital stay				
Length of hospital stay				
Sample, *n*	10,881	2008	8873	0.002^†^
Median (IQR)	13 (10–19)	14 (10–19)	13 (10–19)	

*ICU* intensive care unit, *IQR* interquartile range, *NNS *new-onset neurological symptoms

*Difference between NNS and non-NNS groups

^†^Statistically significant (i.e., alpha set at 0.5)

**Table 5:** Crude and fully adjusted hazard ratio for mortality, ICU admission and respiratory failure comparing patients with NNS vs. patients without NNS

Characteristic	HR (95% CI)	*p* value for association^†^
Mortality		
Crude HR for mortality	1.423 (1.278–1.585)	–
Fully adjusted HR for mortality		
COVID-19 severity*		
Mild	1.660 (1.132–2.434)	0.01^‡^
Severe	1.352 (1.042–1.752)	0.023^‡^
Critical	1.043 (0.920–1.181)	0.511
Confounders		
Hypertension	0.863 (0.781–0.954)	0.04^‡^
Age	1.010 (1.007–1.013)	< 0.001^‡^
Sex (male)	0.959 (0.870–1.058)	0.405
Respiratory failure		
Crude HR for respiratory failure	1.857 (1.670–2.065)	–
Fully adjusted HR for respiratory failure		
COVID-19 severity*		
Mild	1.914 (1.346–2.722)	< 0.001^‡^
Severe	1.614 (1.260–2.068)	< 0.001^‡^
Critical	1.234 (1.089–1.398)	0.001^‡^
Confounders		
Smoker	1.364 (1.889–1.565)	< 0.001^‡^
Hypertension	1.517 (1.355–1.699)	< 0.001^‡^
Diabetes mellitus	1.174 (1.055–1.307)	0.003^‡^
Age	0.999 (0.996–1.002	0.536
Sex (male)	0.852 (0.768–0.945)	0.003^‡^
ICU admission		
Crude HR for ICU admission	1.802 (1.626–1.996)	–
Fully adjusted HR for ICU admission		
COVID-19 severity*		
Mild	1.973 (1.457–2.673)	< 0.001^‡^
Severe	1.831 (1.506–2.226)	< 0.001^‡^
Critical	1.122 (0.985–1.279)	0.084
Confounders		
Smoker	1.443 (1.267–1.643)	< 0.001^‡^
Hypertension	1.692 (1.516–1.889)	< 0.001^‡^
Diabetes mellitus	1.268 (1.144–1.404)	< 0.001^‡^
Age	0.999 (0.996–1.002)	0.630
Sex (male)	0.884 (0.800–0.977)	0.016^‡^

*95% CI* 95% confidence intervals, *NNS* new-onset neurological symptoms, *HR* hazard ratio, *ICU* Intensive care unit

*Interaction term (likelihood ratio test) *p* value = 0.03

^†^Wald’s test

^‡^Statistically significant (i.e., alpha set at 0.05)

#### Mortality

A total of 1702 (15.6%) died in the full cohort; acute respiratory distress syndrome (ARDS) (*n* = 749, 44.0%) and septic shock (*n* = 699, 41.1%) were the typical causes of mortality. Among those who died, a significantly higher proportion of patients was found in the NNS compared to those in the non-NNS group (*p* < 0.001); among those who died due to ARDS (*p* < 0.001), septic shock (*p* = 0.001), and brain herniation (*p* < 0.001), a statistically higher percentage of patients was found in the NNS group. The crude HR for mortality was significantly higher among those in the NNS group by 1.423 (95% CI 1.278–1.585) than those in the non-NNS group (see Fig. 2A). After adjusting for age, sex, and presence of HPN, the risk of mortality differed depending on the disease severity. Among the mild and severe cases, the adjusted HRs remained significantly higher by 1.660 (95% CI 1.132–2.435; *p* = 0.01) and by 1.352 (95% CI 1.042–1.752; *p* = 0.023), respectively, in the NNS group compared to those in the non-NNS group. However, among the critical cases, the adjusted HR for mortality became non-significant (HR 1.043, 95% CI 0.920–1.181; *p* = 0.511).

#### Respiratory failure

There were 1608 (14.8%) patients who had respiratory failure. Pneumonia (*n* = 927, 57.6%) and ARDS (*n* = 868, 54%) were the most common causes of respiratory failure. Among those who had respiratory failure, a higher proportion of patients was found in the NNS compared to the non-NNS group (*p* < 0.001); among those who had a respiratory failure due to pneumonia (*p* < 0.001), ARDS (*p* < 0.001), shock (*p* < 0.001), central neurological cause (*p* < 0.001), and pulmonary edema (*p* < 0.001), a statistically higher percentage of patients was found in the NNS group. The crude HR for respiratory failure was significantly higher among those in the NNS group by 1.857 (95% CI 1.670–2.065) than those in the non-NNS group (see Fig. 2B). After adjusting for age, sex, smoking status, and presence of HPN and DM, the risk of respiratory failure differed depending on the disease severity. Among the mild, severe, and critical cases, the adjusted HRs for respiratory failure were still significantly increased by 1.914 (95% CI 1.346–2.722; *p* < 0.001), by 1.614 (95% CI 1.260–2.068; *p* < 0.001) and by 1.234 (95% CI 1.089–1.398; *p* = 0.001), respectively, in the NNS group than those in the non-NNS group.

#### Duration of ventilator dependence (DVD)

Among those patients needing mechanical ventilation, the overall median DVD was 13 days (IQR 8–20). There was no significant difference in the median DVD between patients in the NNS and non-NNS groups (*p* = 0.612). The crude OR for longer DVD (i.e., ≥ 16 days) was insignificant at 0.979 (95% CI 0.793–1.207; *p* = 0.841). After adjusting for age, sex, and COVID-19 severity, the adjusted OR for longer DVD remained insignificant at 0.954 (95% CI 0.772–1.179; *p* = 0.663).

#### ICU admission

A total of 1740 patients (16.0%) from our full cohort were admitted to the ICU. ARDS (*n* = 956, 54.9%) and shock (*n* = 180, 10.3%) were the most common reason for ICU admission. Among those admitted to the ICU, a higher proportion of patients was found in the NNS compared to the non-NNS group (*p* < 0.001). Similarly, a statistically higher percentage of patients was found in the NNS group among those who were admitted to the ICU due to ARDS (*p* < 0.001), shock (*p* < 0.001), impaired level of consciousness (*p* < 0.001), acute kidney injury necessitating dialysis (*p* < 0.001), treatment-related indication (*p* < 0.001), acute stroke (*p* < 0.001), and cerebral edema (*p* < 0.001). The crude HR was significantly higher among those in the NNS group by 1.857 (95% CI 1.670–2.065) than those in the non-NNS group (see Fig. 2C). After adjusting for age, sex, smoking status, and presence of HPN and DM, the risk for ICU admission differed depending on disease severity. Among the mild and severe cases, the adjusted HRs for ICU admission were still significantly higher by 1.973 (95% CI 1.457–2.673; *p* < 0.001) and by 1.831 (95% CI 1.506–2.226; *p* < 0.001), respectively, in the NNS group compared to those in the non-NNS group.

#### Length of ICU stay (LICUS)

Among those patients admitted to the ICU, the overall median LICUS was 15 days (IQR 9.5–21). There was no significant difference in median LICUS between patients in the NNS and non-NNS groups (*p* = 0.887). The crude OR for longer LICUS (i.e., 16 days) was insignificant at 0.969 (95% CI 0.763–1.229; *p* = 0.793). After adjusting for age, sex, and disease severity, the adjusted OR for longer LICUS remained insignificant at 0.983 (95% CI 0.772–1.252; *p* = 0.892).

#### Length of hospital stay (LHS)

Among the full cohort, the overall median LHS was 13 days (10–19). There was a significant difference in the median LHS between patients in the NNS and non-NNS groups (*p* = 0.002). The crude OR for longer LHS (i.e., ≥ 14 days) was insignificant at 1.037 (95% CI 0.941–1.142; *p* = 0.465). After adjusting for age, sex, and disease severity, the adjusted OR for longer LHS remained insignificant at 1.045 (95% CI 0.947–1.153; *p* = 0.378).

## Discussion

To the best of our knowledge, we presented the largest Philippine cohort study involving 10,881 hospitalized patients with COVID-19 infection with substantial information on their neurological characteristics. Furthermore, we investigated the effects of the presence of neurological manifestations at the onset on clinically relevant outcomes in these patients.

The incidences of at least one new-onset neurological signs/symptoms in the context of COVID-19 infection were extremely wide-ranging, i.e., ~ 12 to ~ 85%. The wide extent of these percentages was based on retrospective and prospective studies from various countries: Austria (Zifko et al. 2021); China (Mao et al. 2020; Xiong et al. 2020) Egypt (Khedr et al. 2021); Iran (Amanat et al. 2021; Ghaffari et al. 2021); Italy (Benussi et al. 2020; Rifino et al. 2021; Travi et al. 2021); France (Kremer et al. 2020); Germany (Fleischer et al. 2021); Mexico (Flores-Silva et al. 2021); Pakistan (Iltaf et al. 2020); Poland (Wnuk et al. 2021); Portugal (Oliveira et al. 2021); South Korea (Kim et al. 2021); Spain (García-Azorín et al. 2021a, b; García-Moncó et al. 2020; García-Azorín et al. 2021a, b; Romero-Sánchez et al. 2020); Turkey (Karadaş et al. 2020; Yuksel et al. 2021); United States of America (Chachkhiani et al. 2020; Eskandar et al. 2021; Frontera et al. 2021; Liotta et al. 2020); multinational (Chou et al. 2021). Systematic reviews with or without meta-analysis (Cagnazzo et al. 2021; Chua et al. 2020; Collantes et al. 2021; Pinzon et al. 2020; Romoli et al. 2020; Tsai et al. 2020; Vakili et al. 2021) and narrative/scoping reviews (Ahmed et al. 2020; Maury et al. 2021; Roy et al. 2021; Shehata et al. 2021; Wenting et al. 2020; Whittaker et al. 2020; Xu et al. 2021; Yachou et al. 2020; Zubair et al. 2020) were also published. Typically described new-onset neurological manifestations of COVID-19 in the literature included anosmia/ageusia, headache, nausea, myalgia, fatigue, dizziness, acute cerebrovascular events, seizures, and altered mental status/encephalopathy. In our cohort study, we showed that approximately one in twenty COVID-19 patients (i.e., ~ 5%) had a new-onset headache, anosmia/hyposmia, and altered sensorium while one in eleven patients (i.e., ~ 9%) had a new-onset neurological disorder. The most common complications were encephalopathy, stroke and seizure events. It is unknown and difficult to determine if the considerable variations in the percentages of neurological manifestations among the studies are due to differences in the susceptibility among the diverse populations or the neuroinvasive potentials of certain COVID-19 variants. However, we theorize that the varying definitions or ascertainment of neurological manifestations, method of data collection, recording/recall bias, sampling error and other biased played a much larger role in these differences.

Among the full cohort in our study, a substantial number experienced respiratory failure (14.8%), were admitted to the ICU (16.0%) and died (15.6%). Of particular concern was that the new-onset neurological symptoms (NNS) in COVID-19 patients significantly increase the risk of poor outcomes such as mortality, respiratory failure and ICU admission compared to those patients without NNS. On the other hand, the presence of NNS was not significantly associated with a longer length of mechanical ventilation, ICU stay, and hospital admission.

Our data showed that the crude HR of mortality in COVID-19 patients with NNS was increased by 42% over those without NNS. With adjustments for confounders and taking into account the disease severity, mortality risk is significantly higher by 66% among mild cases and by 35% among severe cases. Among critically ill individuals, the presence of NNS may seem to be irrelevant to mortality. We believe that other probable, more fundamental factors may be operating as disease severity progresses such as cardiopulmonary and metabolic complications. Compared to the other matched case–control study with 2324 COVID-19 patients done in NYC, the odds of death was significantly increased by 27% in those with neurological manifestations than those without (Eskandar et al. 2021). A large cohort study also conducted in NYC with 4491 COVID-19 patients showed a significant increase in HR by 38% after adjusting for significant confounders, which was comparable to our own estimates (Frontera et al. 2021). In contrast to these studies, our cohort demonstrated that the proportions of COVID-19 patients with NNS who died due to ARDS, septic shock, multi-organ dysfunction syndrome, and brain herniation were significantly higher compared to those without NNS.

It may be intuitive to suspect that a city with a high population density like NYC would yield worse outcomes particularly in patients with COVID-19; however, other underlying factors may be operative particularly in our country where the healthcare system is at higher risk of being overwhelmed (COVID-19): an ongoing public health crisis in the Philippines 2021). In 2018, there were 23 beds per 10,000 individuals in the National Capital Region while the rest of Luzon, Visayas, and Mindanao had only 8.2, 7.8, and 8.3 beds, respectively (Dayrit et al. 2018). The increase in the role of the private sector in healthcare service delivery induces reliance on out-of-pocket healthcare expenses. In 2018, a total of ~ 54% and ~ 12% is supplied by out-of-pocket payment and voluntary health care payment methods, respectively, to the health expenditure financing scheme (Ignacio et al. 2020; Mapa 2019). The deficient medical infrastructure, insufficient healthcare workforce, and the inadequate government action on contact tracing, mass testing, and vaccine rollout remains to be a major problem that may lead to poor outcomes for our patients infected with COVID-19 (COVID-19): an ongoing public health crisis in the Philippines 2021).

In our cohort, we also provided initial and relatively precise proof that NNS in COVID-19 infection significantly increases the HR of developing respiratory failure with significantly different magnitudes of effect in terms of disease severity after fully adjusting for factors; 91%, 61% and 23% in mild, severe, and critical cases, respectively. In addition, our data showed that apart from pneumonia, ARDS, shock and pulmonary edema, a significantly higher proportion of COVID-19 patients with NNS had respiratory failure attributed to central neurological causes compared to those in the non-NNS group. These support clinical evidence on the hypothesis that SARS-CoV-2 may infect the medullary cardiorespiratory centers via the peripheral nerve terminals (e.g., baro- and chemoreceptors) in the pulmonary system through synapse-connected routes (Li et al. 2020; Machado-Curbelo 2020). Histopathological studies of several brain specimens demonstrated evidence of viral infiltration or encephalitic characteristics with neuronal cell loss and axonal degeneration affecting brainstem nuclei and tracts without documented infarction (Jaunmuktane et al. 2020; Younger 2021). In a recent comprehensive systematic review of neuropathologic findings in COVID-19 patients, other possible mechanisms that can potentially contribute to damage in cardiorespiratory centers in the brainstem leading to respiratory failure in COVID-19 infection may involve hypoxic/ischemic changes or territorial infarctions, microglial/astrocytic activation, and reactive gliosis (Pajo et al. 2021).

Furthermore, our study was able to shed light on relevant evidence relating NNS and ICU admission in general. In particular, there was a significantly increased risk (i.e., added 80% risk) for ICU admission in COVID-19 patients with NNS compared to those without NNS. Similar to mortality and respiratory failure outcomes, disease severity was found to be a significant effect modifier for ICU admission; 97% and 83% increased risk in mild and severe cases, respectively. These data suggest that NNS may potentially predict increased utilization of ICU/ICU-comparable settings that may lead to elevated healthcare costs among patients with COVID-19 patients. In addition, our data showed that there were significantly higher percentages of patients in the NNS cohort admitted due to ARDS, shock, impaired level of consciousness, acute kidney injury necessitating dialysis, acute stroke, and cerebral edema compared to those in the non-NNS.

Indeed, most previously published reports were focused on the characterization of NNS in COVID-19. Compared to these precursory studies, our study imparted valuable information from a much larger sample size of COVID-19 patients which provides an advantage of limiting sampling error and allows more precise estimates of the associations. To extend the comprehensiveness of our report, the findings in the neuroimaging and CSF were also provided. Although a number of previous studies provided some insights on the association of neurological manifestations to in-hospital mortality, our study provided new information on the relationship of NNS to specific causes of mortality, to the incidence of respiratory failure and its specific causes, to the need for admission to the ICU and the particular reasons for the admission. Furthermore, our study also revealed data on the associations of NNS with a longer duration of ventilator dependence, a longer length of ICU stay, and longer hospital stay. At present, the relationships between NNS and these other outcomes are insufficiently described in the literature. These outcomes can be also considered clinically useful particularly to estimate the magnitude of the risk of having certain poor outcomes when a COVID-19 patient had NNS compared to those without NNS.

Our study has inherent limitations. The data reflected exposures and outcomes of hospitalized COVID-19 patients; thus, information from patients who were not admitted was not captured by our estimates. Moreover, because only admitted patients were involved in this study, mortality and respiratory failure are expected to be overestimated since substantially more severe and critical COVID-19 patients are admitted to the hospital. There may be certain periods during the collection of data when our healthcare system was overwhelmed. Hence, the availability to admit patients to the hospital or to the ICU as well as the capacity to sufficiently document clinical/neurological features and outcomes of patients by our local admitting neurologists/physicians could have affected our estimated exposures/outcomes. In this cohort study, the comparison in the neurological features and outcomes of patients admitted in the urban and rural hospital setting is not feasible because all the included study sites were located in the urban setting. Other study limitations were non-documentation of their psychiatric symptoms and the treatments employed for their neurological symptoms. Recording bias was intrinsic to this study which could have contributed to underreporting of data. Nevertheless, we were able to obtain large amounts of data from the total enumeration of COVID-19 patients from 37 hospital sites located in various regions of our country. We further theorize that prospective collection of data may probably increase the effect estimates of NNS on mortality, respiratory failure, and ICU admission and may facilitate detection of other relevant confounding variables and effect modifiers affecting their relationship.

Our current study encompassed patients admitted from February to December 2020 only. It may be worthwhile to look into the differences in the neurological features of COVID-19 in 2020 compared to 2021 given the emergence of COVID-19 variants and the effects of vaccination. Previous observational studies, including our current study, provided compelling evidence of the involvement of the nervous system particularly the central nervous system (CNS). Beyond the clinical symptomatology, subsequent studies may focus on the viral staging pathology of the brain as well as documenting neuronal-glial changes in patients with COVID-19-infected CNS regions (Riederer and ter Meulen 2020). Long-term sequelae of previously affected brains by the COVID-19 that could potentially promote or precipitate certain neurodegenerative disorders must be evaluated in future clinical and brain coronavirus-related research (Riederer and ter Meulen 2020).

## Data Availability

Anonymized data not published within this article will be made available by request from any qualified investigator.

## Abbreviations

95% CI: 95% confidence intervals
ARDS: Acute respiratory distress syndrome
COVID-19: Coronavirus disease 2019
DM: Diabetes mellitus
DVD: Duration of ventilator dependence
HPN: Hypertension
HR: Hazard ratio
ICU: Intensive care unit
IQR: Interquartile range
LHS: Length of hospital stay
LICUS: Length of ICU stay
NNS: New-onset neurological symptoms
OR: Odds ratio
RT-PCR: Reverse transcription polymerase chain reaction
SARS-CoV-2: Severe acute respiratory syndrome coronavirus 2

## Declarations

### Technical review

Our protocol underwent technical review from the following institutions: Department of Neurosciences, Philippine General Hospital and College of Medicine, University of the Philippines Manila, from the Cardinal Santos Medical Center, San Juan City, and from the Research Center for Clinical Epidemiology and Biostatistics, De La Salle Medical and Health Sciences Institute, Dasmariñas City, Cavite.

### Protocol registration and publication

Our protocol was registered at the ClinicalTrials.gov website (NCT04386083). The full protocol was published by the *British Medical Journal (BMJ) Open* (see the following article: Espiritu AI, Sy MCC, Anlacan VMM, Jamora RDG. The Philippine COVID-19 Outcomes: a Retrospective study Of Neurological manifestations and Associated symptoms (The Philippine CORONA study): a protocol study. BMJ Open 2020; 10:e040944. Available at: https://bmjopen.bmj.com/lookup/doi/10.1136/bmjopen-2020-040944.

### List of study sites

Asian Hospital and Medical Center, Muntinlupa City; Baguio General Hospital and Medical Center, Baguio City; Cagayan Valley Medical Center, Tuguegarao City; Capitol Medical Center, Quezon City; Cardinal Santos Medical Center, San Juan City; Chong Hua Hospital, Cebu City; De La Salle University Medical and Health Sciences Institute, Dasmariñas City; East Avenue Medical Center, Quezon City; Jose B. Lingad Memorial Regional Hospital, City of San Fernando, Pampanga; Dr. Jose N. Rodriguez Memorial and Sanitarium Hospital, Caloocan City; Jose R. Reyes Memorial Medical Center, Manila; Lung Center of the Philippines, Quezon City; Manila Doctors Hospital, Manila; Medical Center Manila, Manila; Makati Medical Center, Makati City; New Era General Hospital, Quezon City; Northern Mindanao Medical Center, Cagayan de Oro City; Quirino Memorial Medical Center, Quezon City; Ospital ng Makati, Makati City; Perpetual Succour Hospital, Cebu City; Dr. Pablo O. Torre Memorial Hospital (Riverside Medical Center, Inc.), Bacolod City; University of the Philippines—Philippine General Hospital, Manila; Philippine Heart Center, Quezon City; Research Institute for Tropical Medicine, Muntinlupa City; San Lazaro Hospital, Manila; San Juan De Dios Educational Foundation Inc.—Hospital, Pasay City; Southern Isabela Medical Center, Santiago City; St. Luke’s Medical Center, Quezon City; St. Luke’s Medical Center, Bonifacio Global City, Taguig City; Southern Philippines Medical Center, Davao City; The Medical City, Pasig City; University of the East Ramon Magsaysay Memorial Medical Center, Inc., Quezon City; University of Santo Tomas Hospital, Manila; Veterans Memorial Medical Center, Quezon City; Vicente Sotto Memorial Medical Center, Cebu City; Western Visayas Medical Center, Iloilo City; Zamboanga City Medical Center, Zamboanga City.
